# The Role of miR-20 in Health and Disease of the Central Nervous System

**DOI:** 10.3390/cells11091525

**Published:** 2022-05-03

**Authors:** Ivan Arzhanov, Kristyna Sintakova, Nataliya Romanyuk

**Affiliations:** 1 Department of Neuroregeneration, Institute of Experimental Medicine of the Czech Academy of Sciences, 142 20 Prague, Czech Republic; ivan.arzhanov@iem.cas.cz (I.A.); kristyna.sintakova@iem.cas.cz (K.S.); 2 Department of Neuroscience, 2nd Medical Faculty, Charles University, 150 00 Prague, Czech Republic

**Keywords:** microRNA, miR-20a, central nervous system

## Abstract

Current understanding of the mechanisms underlying central nervous system (CNS) injury is limited, and traditional therapeutic methods lack a molecular approach either to prevent acute phase or secondary damage, or to support restorative mechanisms in the nervous tissue. microRNAs (miRNAs) are endogenous, non-coding RNA molecules that have recently been discovered as fundamental and post-transcriptional regulators of gene expression. The capacity of microRNAs to regulate the cell state and function through post-transcriptionally silencing hundreds of genes are being acknowledged as an important factor in the pathophysiology of both acute and chronic CNS injuries. In this study, we have summarized the knowledge concerning the pathophysiology of several neurological disorders, and the role of most canonical miRNAs in their development. We have focused on the miR-20, the miR-17~92 family to which miR-20 belongs, and their function in the normal development and disease of the CNS.

## 1. Introduction

Acute injuries of the CNS, such as spinal cord injury (SCI), traumatic brain injury (TBI), and stroke, make up a significant portion of all injuries worldwide. In the USA alone, 13.5 million people are affected [1]. Historical advances in symptom management have reduced the mortality rate. However, there is still no effective treatment to counteract the long-term functional deficits following injuries. At the same time, neurodegenerative disorders such as Alzheimer’s disease (AD) and Parkinson’s disease (PD) affect millions worldwide, and prevalence is expected to increase as the population ages. In 2010, it was estimated that there were 35.6 million people living with dementia across the globe; these numbers are expected to double every 20 years until 2050 [2]. As with traumatic injuries, there is no effective treatment for neurodegenerative diseases. To increase understanding of these disorders, extensive research in biochemistry, genetics, epigenetics, and classical neurobiology is required.

miRNAs, short non-coding ∼22 ncRNAs that modulate protein expression levels by antagonizing mRNA, are highly expressed in the mammalian CNS. They play crucial roles in virtually every aspect of CNS function, including neurogenesis, neural development, and cellular response to pathological conditions such as inflammation, apoptosis, cell proliferation, and differentiation. Recent studies have shown that miRNAs are dysregulated following a variety of CNS injuries, which has attracted them considerable attention as potential therapeutic targets [1,3,4,5,6]. The ability of miRNAs to regulate cell state and function through the post-transcriptional silencing of hundreds of genes was acknowledged as an important player in the pathophysiology of CNS disorders. MicroRNAs are released as circulating molecules into body fluids such as CSF, blood, and urine. Therefore, they may serve as valuable biomarkers for detecting early onset neurodegenerative disorders. MicroRNAs have the potential to be therapeutic molecules. MicroRNA inhibitors and mimics can be used to target pathologically upregulated and down-regulated miRNAs [7].

In this Review, we provide an overview of the miR-17~92 family, and summarize the role of its members in neurogenesis and neurological disorders. Furthermore, we provide a description of the most common neurological disorders including SCI, stroke, TBI, AD, PD, and the role of canonical miRNAs in their pathophysiology with a focus on miR-20. Finally, we summarize the possible mechanisms of miR-20 involvement in the various physiological and pathological processes in the CNS.

## 2. miR-17~92 Family

**Figure 1:**
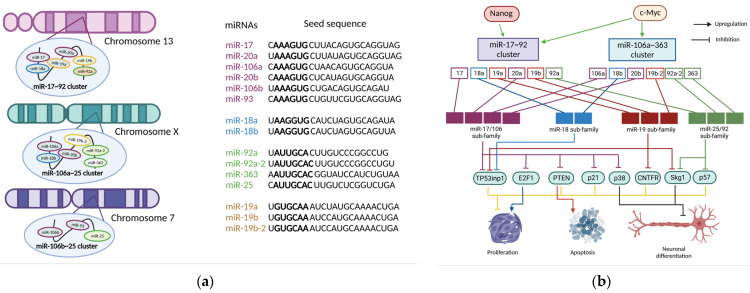
The miR-17~92 family, its gene structure, members, and their role in the normal development of the nervous system. (**a**) Sequences of cluster members miR-17/92 and its two paralogs miR-106a/363 and miR-106b/25. The “seed” in each case is bold. (**b**) Members of the miR-17~92 family inhibit the expression of their multiple targets, resulting in increased proliferation, accelerated neuronal differentiation, and inhibited apoptosis. miRNAs, together with target genes, function as a key regulator of ontogenetic and adult neurogenesis. This image was created using BioRender (accessed on 30 March 2022).

microRNA-20a/b belongs to the miR-17~92 family. The miR-17~92 cluster is a representative example of a polycistronic miRNA gene [8]. It is located in the 13q31.3 region of human chromosome 13, and produces seven individual mature miRNAs: miR-17-3p, miR-17-5p, miR-18a, miR-19a, miR-20a, miR-19b, and miR-92a [8,9,10,11,12]. It has two mammalian paralogs; miR-106b-25 (located on human chromosome 7) and miR-106a-363 clusters (located on the X chromosome). The miR-106b-25 cluster encodes miR-106b, miR-93, and miR-25; the miR-106a-363 cluster encodes miR-106a, miR-18b, miR-20b, miR-19b-2, miR-92a-2, and miR-363 [8,11,13,14]. Based on the described region sequences, the miR-17~92 family is divided into four subfamilies: the miR-17 family (miR-17, miR-20a/miR-20b, miR-106a/miR-106b, and miR-93), the miR-18 family (miR-18a/miR-18b), the miR-19 family (miR-19a/miR-19b), and the miR-25 family (miR-25, miR-92a, and miR-363). The structure of this family, its origin, possible functions, and mechanisms of regulation, are shown in Figure 1.

Initially, the miR-17~92 family was considered to be oncogenic, and it was later shown to trigger various physiological and pathological processes. The role of this miR family and its members in neurogenesis and neurological disorders is described in more detail in Xia et al. [15]. Here we summarize only some of the salient points.

Emerging evidence has implicated the miR-17~92 family in the regulation of neurogenesis by facilitating neural stem cell (NSC) proliferation, suppressing NSC differentiation, and inhibiting apoptosis [10,16,17,18,19,20,21]. The miR-17~92 family achieves its function through targeting various anti-neural or anti-proliferative genes, including phosphatase and tensin homolog (PTEN), Tp53, inp1, and p21 [16]. The general overexpression of both the miR-17~92 family and miR-106b~25 cluster in particular promotes the generation of neurons from NSCs. This suggests that the miR-17~92 family can shift the differentiation of NSCs towards neuronal lineage [22].

**Figure 2:**
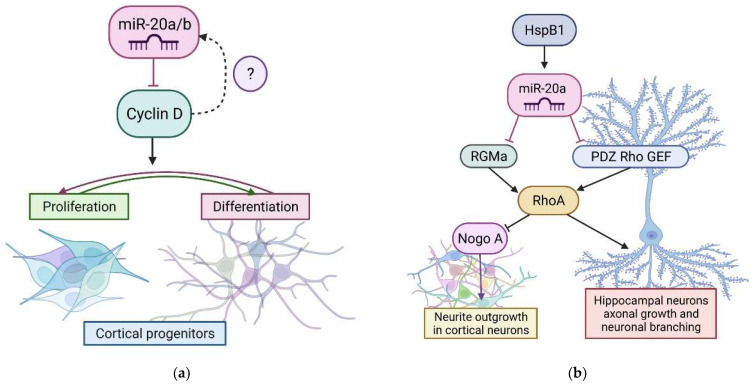
The role of miR-20 in the normal development of the nervous system. (**a**) miR-20a/b regulates developmental stage of cortical neurons by targeting cyclin D1. This regulation underlines fail-safe mechanism. (**b**) Expression of miR-20a can be stimulated by heat shock protein B1 (HspB1), which enhances neurite outgrowth in cortical neurons, and axonal growth and neuronal branching in hippocampal neurons. This image was created using BioRender (accessed on 30 March 2022).

The special role of miR-20 in normal development is disclosed in Ghosh et al. [23]. The authors demonstrated that miR-20a/b, together with miR-23a, regulate the developmental-stage-specific mean and variance of cyclin D1 protein level in a feedback regulatory network (Figure 2a). This regulation underlies the failsafe mechanism that allows cortical progenitors to make decisions about proliferation or differentiation. A deficiency of these miRNAs increases the dynamic range of expression as well as the intermediate expression level of cyclin D1, and impairs the balance between progenitor proliferation and differentiation. Although the components of the miR-20a/b-cyclinD1 network may regulate each other, the precise control of cyclinD1 expression is more complex and requires further study.

Members of the miRNA-17~92 cluster are also highly expressed in oligodendrocytes and their precursors. The conditional deletion of the miR-17~92 cluster in oligodendrocytes using Cnp^+/Cre^ mice was shown to reduce Olig2-positive cells to approximately 25% of the controls at P0 [24]. The microRNA-17~92 cluster has a role in regulating oligodendrocyte proliferation, and its absence leads to a reduction in the number of oligodendrocytes. Additionally, the overexpression of miR-17 and miR-19b in the oligodendroglial cell line Oli-neu increased the number of OLs [25]. According to a bromodeoxyuridine assay, miR-19b-transfected cells exhibited significantly increased OPC proliferation rates when compared to the control miR-transfected cells [25]. These data suggest that the miRNA-17~92 cluster members accelerate OPC proliferation, whereas miRNA-219 and miRNA-338 mitigate it.

miR-20a participates in the regulation of neurite growth during the normal development of cortical neurons. Sun et al. [26] found that the heat shock protein B1 (HspB1), prevents the inhibitory effects of Nogo-A on neurite growth in cortical neurons (Figure 2b). HspB1 belongs to a family of 10 small heat shock proteins that share an α-crystallin domain. These proteins function as molecular chaperones to maintain proteins in a folding-competent state, and serve as key regulators of intermediate filament and microtubule networks [27]. HspB1 is found in most neurons of the CNS and is required for neuronal survival [28]. Researchers demonstrated that HspB1 enhances the expression of a group of miRNAs, including miR-20a, miR-128, and miR-132. Two of these miRNAs, miR-20a and miR-128, can inhibit translation by binding to the 3′UTR promoter region of Rho guanine nucleotide exchange factor (PDZ–RhoGEF) mRNA. It was suggested that HspB1 regulates Ras homolog gene family member A (RhoA) activity through modulation of PDZ–RhoGEF levels. This is achieved by translational control through the enhanced expression of specific miRNAs (miR-20a and miR-128). The regulation of RhoA activity by translational silencing of PDZ–RhoGEF may be the mechanism through which HspB1 is involved in the regulation of neurite growth [26].

Another research group experimentally confirmed miR-20a-5p as a repulsive guidance molecule A (RGMa) upstream regulator. miR-20a-5p regulated RGMa, which in turn regulated RhoA—namely, miR-20a-5p, RGMa, and RhoA, which are all part of the same signaling pathway [29]. Furthermore, in primary hippocampal neurons, the miR-20a-5p/RGMa/RhoA pathway regulated axonal growth and neuronal branching. Given the essential role of Nogo-A and RhoA in several pathologies of the nervous system, the described metabolic pathway must be observed not only in normal development, but also in pathological conditions.

In adults, miR-17~92 influences neurogenesis by regulating genes in the glucocorticoid pathway, especially serum and glucocorticoid-inducible protein kinase-1. Jin et al. [30] used a mouse model to demonstrate a link between this effect and anxious and depressive behavior. MicroRNA-17~92 deficient mice exhibited mood and anxiety disorders, while miR-17~92 overexpressing mice showed anxiolytic and antidepressant behavior. Furthermore, miR-17~92 expression in the adult mouse hippocampus responds to chronic stress, and miR-17~92 rescues proliferation defects induced by corticosterone in hippocampal neural progenitors [30].

Due to its importance in the regulation of neurogenesis, the miR-17~92 family is widely involved in the pathogenesis of neurobiological disorders. Under ischemic conditions in vitro, miR-25 positively regulates adult NSC proliferation in the subventricular zone (SVZ), providing evidence of miR-17~92 family involvement in NSC proliferation in vivo [31]. In addition, the miR-106b~25 cluster also regulates the proliferation of adult NSCs [20]. Moreover, it was demonstrated by Xin et al. [32] that this cluster acts upon the phosphatase and tensin homolog, and triggers the PI3K/Akt/mTOR signaling. Its downstream effector proteins promote neurite remodeling, oligodendrogenesis, axonal growth in primary cortical neurons, cell proliferation, and differentiation, leading to improved functional recovery following stroke in rats [32].

The decrease in miRNA expression levels of the miR-17~92 family during neurogenesis suggests that this family is under precise and integrated regulation. Nanog is the first transcription factor to be associated with the upstream regulatory region of the miR-17~92 family, and maintains high levels of transcription of the latter. In addition, the promoter of the miR-17~92 family can also be occupied by other transcription factors including c-Myc, E2F1, and C/EBP-β in tumor cells [33,34]. Although direct binding of c-Myc to the miR-17~92 encoding gene in NSCs remains unproven, the upregulation of c-Myc increases the expression levels of the miR-17~92 family miRNAs, and therefore modulates neurogenesis [35]. Moreover, both E2F1 and C/EBP-β are expressed in NSCs and their expression levels decrease during neuronal differentiation, which positively correlates with the expression patterns of the miR-17~92 family [36,37]. Furthermore, it has been suggested that because miR-17 and miR-20 target E2F1, which can act as a pro-apoptotic molecule, miR-17~92 could shift the balance from apoptosis to proliferation [38].

## 3. Spinal Cord Injury

The pathophysiology of spinal cord injury (SCI) is a complex intermingled set of events, responses, mechanisms, and processes affecting the nervous, vascular, and immune systems that develop during the months following the initial injury. Detailed descriptions can be obtained in different reviews [39,40,41,42,43]. A brief description of the pathophysiology is provided in the following paragraphs.

### 3.1. Primary Phase

The primary injury phase of SCI involves an initial mechanical insult, which results in/involves either contusion or compression. The damage to upper motoneurons leads to hyperreflexia, hypertonia, and muscle weakness. In contrast, insults to lower motoneurons cause hypotonia, hyporeflexia, and muscle atrophy. Local events include axon severing, membrane rupture and the death of neurons, glia, and endothelial cells. Mechanical trauma causes intraparenchymal hemorrhage and, consequently, the disruption of the blood–spinal cord barrier together with edema and swelling of the spinal cord [44]. Vasospasm and thrombosis result in hypoxia, ischemia, and increasedneural cell death. In the case of severe trauma, hypoxia (together with ion shifts inside and outside the neuron) could cause a temporal switch off of the spinal cord function at and below the injury site, known as spinal shock.

### 3.2. Secondary Phase

The secondary phase of SCI develops within minutes of the primary injury and can last for weeks to months. It can be characterized as several interrelated damage processes including vascular alterations, biochemical disturbances, and cellular responses. These processes lead to an inflammatory response and cell death, which in turn causes a significant expansion of the damaged area. The acute phase begins immediately after the primary spinal cord injury. It disrupts the balance of ion levels, increases excitotoxicity, and the formation of free radicals. Vascular alterations resulting from hemorrhage and ischemia are central constituents of the secondary injury cascade. Reduced perfusion of the spinal cord due to vasospasm and hypotension is followed by a period of reperfusion, which increases the production of oxygen- and nitrogen-derived free radicals (superoxide, hydroxyl radicals, nitric oxide (NO), peroxynitrite); these are already being produced during the period of ischemia [41]. Astrocytes and microglia are activated and together with cells of the immune system produce pro-inflammatory cytokines such as interleukins 1β (IL-1β) and 6 (IL-6), and tumor necrosis factor α (TNF-α). All of these events lead to the development of edema, and contribute to the further development of inflammation [39,42,45].

Mitochondrial dysfunction, associated oxidative stress, and decreased ATP levels, along with the dysregulation of autophagy, result in neuronal death [43]. An active process of programed cell death (apoptosis) increases the secondary damage after trauma to the spinal cord. Apoptosis continues for weeks after the initial trauma. Spinal neurons typically succumb—mostly to necrosis or excitotoxic damage, but also occasionally to apoptosis—within 24 h after SCI. By contrast, oligodendrocytes undergo apoptosis in two distinct phases: an early acute phase lasting for the first 24–48 h, and a later subacute phase that can last up to several weeks after the insult. Anti-apoptotic and pro-apoptotic genes undergo significant changes in expression, including the activation of caspase-3, Bax, and Bak-1 in the first week after SCI, then later the activation of protective phosphatidylinositol 3-kinase (PI3K) and signal transducer and activator of transcription 3 (STAT3) and suppression of pro-apoptotic glycogen synthase kinase 3 (GSK-3) [46,47]. Neuronal apoptosis is mainly mediated by proteins of the Bcl-2 family, including pro-apoptotic members of the BH3 family and anti-apoptotic members such as myeloid cell leukemia sequence-1 (Mcl-1) [40,48]. Axons are demyelinated, neurons and oligodendrocytes die by apoptotic cell death, and further cell necrosis occurs.

Another consequence of SCI is the formation of a glial scar, which impedes axonal regeneration. After CNS injury, astrocytes respond with a characteristic hypertrophic response accompanied by an increased production of intermediate filaments, such as glial fibrillary acidic protein—a process termed reactive astrocytosis or astrogliosis. In the hours following CNS injury, these astrocytes, due to their large cell bodies and processes, join together. Over time, more cell types, including microglia, macrophages, leptomeningeal cells, and dividing progenitor cells, are recruited, culminating in the formation of a glial scar. This structure poses a problem for axonal regeneration: it acts as a physical barrier and accumulates molecules, such as chondroitin sulfate proteoglycans, that inhibit axonal outgrowth [43].

In the months and years that follow the injury, the SCI becomes chronic. Axons are demyelinated, neurons and oligodendrocytes die by apoptotic cell death, and further cell necrosis occurs. A cystic cavity and a glial scar are formed [42]. Partial remyelination gradually occurs, but mostly replaces oligodendrocytes. These oligodendrocytes may be of either progenitor or endogenous NSC origin. Progenitor oligodendrocytes differentiate into myelinating oligodendrocytes. In this way, they remyelinate axons that have survived or regenerated. Myelin loss and the alteration of ion channel function can lead to changes in the surviving neurons and dependent networks, leading to chronic pain and/or spasticity [43].

Several studies have analyzed the miRNA expression and function in SCI animal models [49,50,51,52]. A microarray study of a contusion model of SCI in rats found that, when compared to the baseline, over 35% of the miRNAs expressed in the spinal cord were significantly affected within the first 7 days following injury [50]. The affected miRNAs were differentially regulated, either demonstrating a sustained increase in expression after injury or a sustained decrease. Interestingly, as the injury response progressed, the number of miRNAs that were downregulated gradually increased, whereas the number of upregulated miRNAs remained constant. These data, along with another separate analysis that assessed changes in mRNAs in a similar model of SCI [53], suggest that a negative correlation exists between miRNA and mRNA expression patterns in SCI.

MicroRNAs serve as important regulators in virtually every pathophysiological process of SCI. The most canonical miRNAs and their validated targets are summarized in Table 1. miR-21 is most likely one of the more important and well-studied of these. The overexpression of miR-21 protects neural cells from death by repressing the expression of pro-apoptotic molecules Fas ligand [54], tropomyosin alpha-1 chain (TPM1) and PTEN [55], and programmed cell death protein 4 (PDCD4) [56]. miR-21 can also trigger mechanisms of secondary cell death by reduction of the expression of voltage-gated (L-type) Ca_2+_ channels [57]. However, probably the most important role of miR-21 is the regulation of astrogliosis—another hallmark of the cellular response to CNS injury. Its expression increases in a time-dependent manner following SCI [49,50,52], and miR-21 is highly expressed in astrocytes during the chronic stage [1]. The BMP–BMPR–miR-21 axis is suggested to be a key regulator of astrocytic hypertrophy and glial scar progression after SCI [5].

Other key players in SCI pathophysiology are miR-181 [58] and miR-125b [59], the suppression of which leads to increasing levels of the pro-inflammatory and pro-apoptotic factor TNF-α. The increased levels of cytokines IL-6 and IL-1β during the first days after injury correlate with a reduced expression of its regulators let-7a [60], miR-181a [59,60], miR-30b-5p, and miR-30c [50,52]. Simultaneously, pro-inflammatory cytokines lead to the activation of the NF-κB signaling pathway, which is also under the regulation of miR-9 and miR-199 [52]. Theis et al. [61] showed that miR-133b contributes to spinal cord regeneration through the downregulation of its target RhoA, a small GTPase that inhibits axonal growth. The downregulation of miR-124, miR-34a, and miR-219 after SCI may also contribute to to a decrease in the regenerative capacity of axons of spinal cord neurons [62].

**Figure 3:**
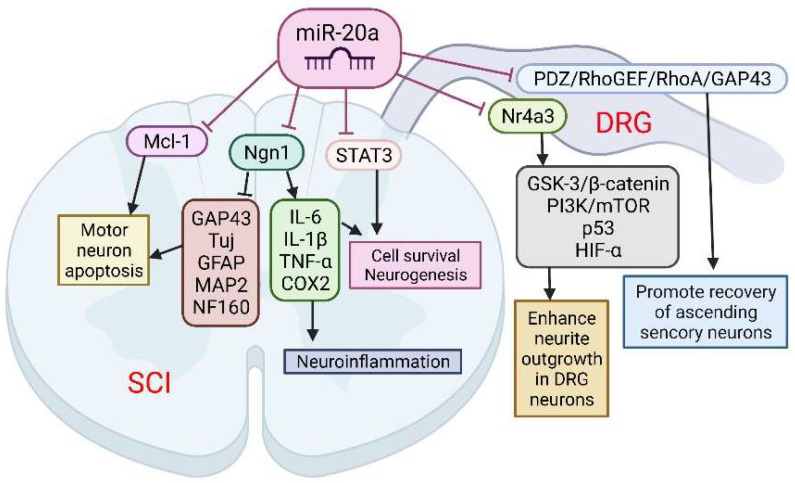
The role of miR-20a in spinal cord injury (SCI) and spinal cord dorsal lesions. SCI led to increased miR-20a expression and targeting of several proteins simultaneously: neurogenin 1 (Ngn1), Mcl-1, and STAT3. Suppression of these proteins has been implicated in several pathological events characteristic of the second phase of SCI: apoptosis of motor neurons, cell death, and neuroinflammation. On the other hand, miR-20a may increase neurite outgrowth in DRG neurons and promote recovery of ascending sensory neurons by interfering with Nr4a3 and PDZ–RhoGEF expression. This image was created using BioRender (accessed on 30 March 2022).

The role of miR-20a in SCI was implicated following observation that its expression was up-regulated for at least 1 week after SCI. This was confirmed by several microarray studies [50,51,52]. Overall, miR-20a has been shown to play a crucial role in the pathophysiology of SCI. Jee et al. [63] showed that the abnormal expression of miR-20a is able to induce secondary injury in adult mice subjected to a transection model of SCI (Figure 3). The authors injected miR-20a into the surgically exposed spinal cord, and demonstrated that miR-20a induced apoptotic neural cell death after 2 days of infusion [63]. Mechanistically, miR20a was found to target neurogenin 1 (Ngn1), a transcription factor that is involved in neuronal differentiation and specification [86]. Ngn1 plays a key role in maintaining cell survival, self-renewal, and neurogenesis, in both the normal and injured spinal cord [63]. Importantly, the inhibition of Ngn1 by siNgn1 in the normal spinal cord of rats has a similar effect on the traumatic injury of the spinal cord. The infusion of siNgn1 into the spinal cord for 3 days even resulted in complete paralysis. The inhibition of Ngn1 in the normal spinal cord significantly increased the cytotoxic effect on motor neurons, as well as on the physiological microenvironment, by increasing the expression of IL-6, caspase-3, IL-1β, TNF-α, and cyclooxygenase 2 [63].

Furthermore, the inhibition of miR-20a activity in vivo using a miRNA inhibitor has been shown to effectively control motor neurons by up-regulating several neuro-specific proteins, including Tuj, microtubule-associated protein 2ab (MAP2ab), neurofilament 160 (NF160), myelin binding protein (MBP), glial fibrillary acidic protein (GFAP), and growth associated protein 43 (GAP43). It was also confirmed that the infusion of antisense miR20a resulted in the reactivation of STAT3/Jak2/ERK1/2, along with increased PI3K/Akt phosphorylation. At the same time, it led to a down-regulation of apoptotic cell death signals, such as Bax and cytochrome C. Interestingly, treatment with exogenous Ngn1 ameliorated the traumatic damage otherwise observed after traumatic SCI. This led to a significant increase in mature neuronal markers, such as TuJ, NF160, MAP2ab, and MBP-positive myelin, while the expression of inflammation inducing factors remained unchanged. These findings validate the functional relevance of the interaction between miR-20a and Ngn-1, but miR-20a has also been shown to target STAT3 (a key mediator in the SCI response) [87], suggesting that miR-20a can affect the response to SCI via multiple pathways.

The authors suggest that the inhibition of miR-20a in traumatic SCI significantly reduces apoptosis and functional deficits through the up-regulated expression of the major target gene, Ngn1. Additionally, a functional deficit in miR-20a-inhibited or Ngn1-infused SCI animals was significantly ameliorated, as was tissue damage, and hindlimb reflexes were recovered.

Further evidence for the involvement of miR-20a in the regulation of apoptosis pathways after SCI was demonstrated by Liu et al. [50]. The authors have shown the up-regulation of miR-20a in a contusion model of SCI, which is consistent with previous studies [50,51,52]. They identified myeloid cell leukemia sequence-1 (Mcl-1) protein as a downstream target regulated by miR-20a. Mcl-1, an anti-apoptotic Bcl-2 family member, is required for neural precursor survival and the regulation of injury-induced neuronal cell death [40]. The inhibition of miR-20a led to an increase of Mcl-1 expression in experiments in vitro and in vivo, in parallel with the decreasing pro-apoptotic protein caspase-3. This finding contributes to the concept of a multifunctional role for miR-20 in SCI response.

Among the many consequences, SCI also causes sensory dysfunctions such as paresthesia, dysesthesia, and chronic neuropathic pain. Sensory neurons in the L4-L6 dorsal root ganglia (DRGs) extend axons to form the sciatic nerve along with motor axons. DRG neurons are one of the exceptional mature neurons whose axons can regenerate after injury. Two groups sought to elucidate the role of miR-20a in the axonal outgrowth of primary sensory neurons, and spinal cord dorsal column lesion (SDLC). Wang et al. [64] suggested that the effect of miR-20a on axonal regeneration is realized via the PDZ–RhoGEF/RhoA/GAP43 axis (Figure 3). The transfection of miR-20a lowered the expression of the key downstream protein, GTP-RhoA, and facilitated the DRG neuron axon regeneration in an inhibitory environment imitated by Nogo-A-Fc. The axon length in miR-20a and Nogo-A-Fc group was similar to that in the control group, and longer than that in Nogo-A-Fc group in vitro. In vivo regulation of miR-20a altered miR-20a–PDZ–RhoGEF/RhoA/GAP43 axis expression, and promoted the recovery of ascending sensory function post-SDCL [64].

According to the findings of Zhao et al. [65], another mechanism for realizing the beneficial effect of miR-20a on the axon regeneration of DRG neurons is the targeting of the Nr4a3 protein. Nr4a3 is a member of the Nr4a family, which has been reported to play important roles in neuronal diseases and cancers via divers’ pathways, such as GSK3/b-catenin, PI3K/mTOR, P53, and HIF-a. The over-expression of miR-20a enhanced neurite outgrowth in DRG neurons in vitro, and axonal regeneration after injury in vivo. In addition, Nr4a3 suppression mimicked the up-regulating effect of miR-20a on axonal regeneration in DRG neurons [65].

## 4. Stroke

Stroke is defined by the World Health Organization as a clinical syndrome of the rapid onset of focal (or global) cerebral deficit, lasting more than 24 h or leading to death, with no apparent cause other than of vascular origin. Stroke is the cause of approximately 10% of deaths worldwide, and is the second most common cause of death in the developed world. Many of the patients that survive the stroke itself require long-term health care [88,89]. Age is a strong factor contributing to the mortality and poor recovery of patients and, more importantly, age affects the susceptibility to stroke depending on patients’ gender. While earlier in life the risk of ischemic stroke is higher in men, stroke becomes more common in older women [90].

Stroke is classified into two major types: ischemic (around 85% of cases) and hemorrhagic. Ischemic stroke is caused by the obstruction of the blood vessels in the brain. This leads to the formation of a thrombus (thrombotic strokes) or an embolus (embolic strokes). A hemorrhagic stroke is caused by an artery rupture within (intracerebral hemorrhage) or on the surface (subarachnoid hemorrhage) of the brain [91,92]. The interruption of blood flow results in a lack of glucose and oxygen. This leads to the death of neurons that are very sensitive to glucose and ATP deficiency. The area with severe hypoperfusion is known as the ischemic core, around which there is a less hypoperfusion area (known as the penumbra). While neurons in the ischemic core die, cells in the surrounding area are still metabolically active for some time and, depending on the environment, either survive or die. Stroke-caused neuronal death is complex. Many molecular mechanisms contribute to excitotoxicity, oxidative stress, and neuroinflammation, which lead to microglia activation, followed by infiltration of the immune cells due to blood–brain barrier (BBB) disruption. Neuroinflammation plays a key role in both brain damage and brain repair [93]. Most importantly, the extent of permanent damage following a stroke corresponds to the duration of ischemia, and restoration of the blood flow is critical for the prognosis [94].

Several attempts to investigate global profiling of miRNA changes after ischemic stroke were performed during recent years [4,66,95,96]. These all demonstrate that stroke substantially alters the expression profile of miRNAs. The specific modulation of the miRNA expression pattern was observed in both the brain and circulating blood during cerebral ischemia [4,95,97]. Interestingly, the miRNA expression pattern correlates with the extent of the infarct area, allow us to distinguish between different etiologies, and predict the clinical outcome [97,98]. However, less attention, compared to SCI, was paid to miRNA expression changes, which occur in the nervous tissue.

According to microarray studies, between 19–25% of miRNAs were dysregulated within 3 days of reperfusion time [4,97]. Ontological analyses predicted that the targets of the dysregulated miRNAs were involved in angiogenesis, hypoxia, endothelial cell regulation, and the immune response—pivotal pathophysiological processes of ischemic stroke [97]. As well as in SCI, miRNAs play an important role in regulating such processes. In particular, a recent study showed that miR-145 is upregulated and responsible for the translational inhibition of superoxide dismutase-2 in the hypertensive rat brain after stroke [4]. miR-497 has been reported to promote ischemic neuronal death in vitro and in vivo by the direct inhibition of anti-apoptotic genes bcl-2 and bcl-w [68]. In addition, miR-15a expression is significantly increased in cerebral vascular endothelial cell cultures after ischemic insults, and plays a causative role in the regulation of apoptosis by direct targeting bcl-2 in ischemic vascular injury in vitro [69]. On the other hand, miR-320a [70] and miR-21 [54] have been shown to protect neurons from ischemic death by targeting water channel modulators, aquaporins, and the Fas ligand gene, respectively. Several other studies from different groups have also documented that the direct modulation of miR-23a [99], miR-181 [100], miR-29b [101], and let-7f [102] may provide a neuroprotective role in rodent experimental stroke models. Interestingly, stroke also leads to alterations in miRNA expression in neural progenitor cells of the SVZ, and miR-124a in particular mediates stroke-induced neurogenesis by targeting the JAG-Notch signaling pathway [77].

The analysis of predicted and proved miRNA targets that are modulated following SCI and stroke indicates that some of the targeted pathways—such as apoptosis, inflammation, cell proliferation, and differentiation—are shared between these injures. However, close inspection reveals that a given cellular function is often regulated by a different set of miRNAs in each injury. The regulation of neuronal differentiation, for example, is affected in the various CNS injuries, but different miRNAs and gene targets have been implicated in each. As detailed above, miR-20a is upregulated after SCI and targets the pro-neural gene Ngn1 [63]. However, in stroke, miR-124, which promotes neuronal differentiation of neural progenitor cells by targeting Jag1 mRNA, is downregulated [67]. The ability of a single miRNA to have the opposite function in different systems is a frustrating aspect of miRNA biology. It illustrates that miRNA communication is cell context-dependent.

This fact also correlates with the observation that miR-20a expression is altered differently after brain injury when compared to SCI. According to [95] miR-20a is downregulated during the early ischemic phase (24 h) in the blood and brain of rats subjected to transient focal ischemia, whereas Dharap et al. [4] demonstrated that miR-20a was significantly (4.1 fold) upregulated 3 days after stroke in a rat transient middle cerebral artery occlusion model. These timepoints have to be considered in relation to miR-20a as a therapeutic target.

**Figure 4:**
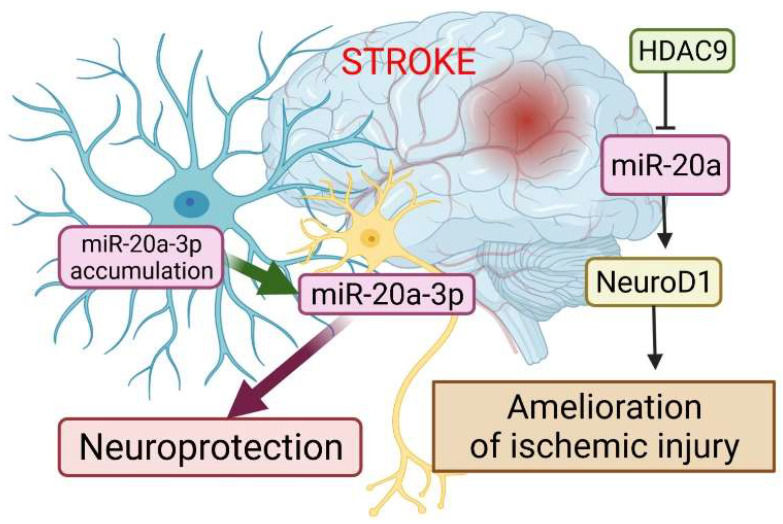
The role of miR-20a in the pathophysiology of stroke. The dysregulation of miR-20a after stroke has a more complex character. Astrocytes are the cells that upregulate miR-20a-3p after stroke; this miRNA is then specifically transferred to neurons or other neural cell types to provide neuroprotection. Another way to alleviate ischemic stroke is to suppress miR-20a by HDAC9 by enriching its promoter, which in turn allows activation of Neuro D1. This image was created using BioRender (accessed on 30 March 2022).

A very recent study has revealed the essential role of miR-20a-3p in the pathophysiology of stroke, and highlighted its therapeutic potential (Figure 4). Branyan et al. [103] used well-known age and sex differences in stroke outcomes to identify miRNA with neuroprotective potential. A comprehensive miRNA screening showed that miR-20a-3p was significantly upregulated in the astrocytes of adult female rats, which typically have better stroke outcomes, while it was profoundly downregulated in the astrocytes of middle-aged females and adult and middle-aged males, groups that typically have more severe stroke outcomes. In vitro studies have shown that miR-20a-3p treatment alters mitochondrial dynamics in both primary human neurons and astrocytes.

Using a tetracycline-induced recombinant adeno-associated virus construct, the researchers provided two delivery methods for miR-20a-3p: astrocyte-specific (downstream a glial fibrillary acidic protein promoter) and neuron-specific (downstream of a neuron-specific enolase promoter). The authors demonstrated that the conditional elevation of astrocyte-specific miR-20a-3p improves survival and stroke-induced sensory motor performance, although it had no effect on infarct volume. Moreover, neuron-specific miR-20a-3p was sufficient to significantly improve infarct volume and sensory motor function. It has been suggested that astrocytes are the cells that upregulate miR-20a-3p after stroke; this miRNA is then specifically transferred to neurons or other neural cell types to provide neuroprotection. A unique aspect of this study is the finding that intravenous injections, which are a therapeutically tractable treatment route, with miR-20a-3p mimic significantly improved stroke outcomes at 4 h after middle cerebral artery occlusion (MCAo), including infarct volume and sensory motor performance. This improvement was not observed when miR-20a-3p was given immediately or 24 h after MCAo, identifying a unique delayed therapeutic window. Overall, these data provide new insights on the neuroprotective role of miR-20a-3p, and characterize several key pathways by which it can improve stroke outcomes [103].

Additional evidence for the involvement of miR-20a in the development of ischemic stroke has been demonstrated by Zhong et al. [71]. Colleagues validated the relationships among histone deacetylases 9 (HDAC9), miR-20a, and its well-known target NeuroD1. HDAC9, a chromatin-modifying enzyme, is widely expressed in brain tissues and plays an important role in the development and maintenance of the nervous system, and is highly upregulated after ischemic injury of the brain [104,105,106]. HDAC inhibitors have shown robust neuroprotection in cerebral ischemia-induced brain injury, which may involve multiple mechanisms, such as activated microglia-mediated inhibition of cerebral inflammation [107]. Zhong et al. [71] showed that HDAC9 downregulated miR-20a by enriching its promoter region, while the silencing of HDCA9 promoted miR-20a expression. miR-20a targeted NeuroD1 and down-regulated its expression. NeuroD1 has been reported as a critical regulator of neuronal development, which is beneficial for stroke recovery. In this study, its downregulation was accompanied by inhibited apoptosis in oxygen-glucose derivate (OGD) neurons. Considering miR-20a targets several proteins involved in the apoptosis pathway, its mechanism of inhibition may be more complex and requires further investigation [71].

Therefore, the silencing of HDAC9 diminished OGD-induced neuronal apoptosis and inflammatory factor release in vitro, as well as ischemic brain injury in vivo, by regulating the miR-20a/NeuroD1 signaling. Overall, this study revealed that HDAC9 silencing could retard ischemic brain injury through the miR-20a/NeuroD1 signaling.

## 5. Traumatic Brain Injury

The acronym TBI refers to traumatic brain injury, defined as “an alteration of brain function, or evidence of brain pathology, that is caused by an external force” [108]. TBI is estimated to become the third leading cause of permanent disability and mortality worldwide [109]. The severity of TBI is determined by the characteristics of the impact and by subsequent complications, such as intracranial pressure or hemorrhage. Similar to SCI, TBI consists of primary and secondary phases and has a very complex pathology with multiple spatially and temporally specific injury mechanisms involved, some of which persist for days or even weeks after the injury itself. The primary injury is caused by mechanical impact, which causes physical damage to the CNS and brain tissue. This leads to the necrotic cell death of neurons and other CNS cell types. Primary injury is followed by secondary injury, a cascade of many molecular and pathophysiological processes such as edema, excitotoxicity, oxidative stress and mitochondrial dysfunction, apoptotic cell death, disruption of BBB, and inflammation. All of these processes contribute to tissue damage. The disruption of BBB enables leukocyte infiltration, exacerbation of edema and cell death. The immune response during the primary phase has a neuroprotective effect, but in the secondary phase promotes further damage and neuroinflammation. Many kinds of chemokines and cytokines are produced [110,111,112,113,114,115].

TBI is usually classified according to the site of injury into open-head and closed-head types, the difference being whether or not the dura is damaged [116]. Furthermore, TBI can be typified into three levels: mild, moderate, and severe [117] based on structural imaging information, alteration of consciousness/mental state (AOC) and Glasgow Coma Scale (GCS).

MicroRNA array and bioinformatic approaches quantified the miRNA expression level and delineated their functions. Several studies revealed the significant upregulation of up to 66 miRNAs, and up to 92 miRNAs were downregulated in the hippocampus and/or cortex of experimental animals with TBI during the first week after injury [118,119,120]. Significant decreases in neuronal miR-107 expression were detected in the CA1 and CA3 of the hippocampus in vivo 1 day after TBI [121]. This was accompanied by the augmentation of granulin (GRN) expression, which is a valid target of miR-107 and acts as a neuronal growth factor to regulate neuronal growth and differentiation, and further promotes neural function recovery [72]. miR-21 expression in the rat hippocampus peaked 3 days post-TBI and returned to near sham levels 15 days after severe TBI [118,122]. The upregulation of miR-21 levels may alleviate BBB leakage and lead to a better neurological outcome after TBI via an improvement in long-term neurological function, decreasing injured brain volume and reducing brain edema [74,123,124]. The miR-34a/Notch1 pathway plays an important role in the regulation of NSC differentiation and proliferation after severe TBI [73]. Decreased miR-34a may improve cognitive function [123]. miR-144 was consistently upregulated in the hippocampus at all the time points (1 h and 1, 3, 5, and 7 d) after experimental TBI. miR-144 may contribute to the alleviation of TBI-induced cognitive dysfunction by targeting Cask and nuclear factor erythroid 2-related factor 2 (NRF2) proteins. Rapid miR-23a and miR-27a downregulation was detected in a mouse cortex from 1 to 24 h after moderate experimental TBI, and the expression levels of these two miRNAs gradually returned to normal levels 72 h post injury. The reductions in these two miRNAs promoted cellular apoptosis in injured cerebral cortex via the activation of pro-apoptotic Bcl-2 proteins, and the administration of a miR-23a mimic significantly reduced cortical lesion volume after TBI and neuronal cell loss in injured hippocampus [75]. However, when considering the role of microRNAs in the pathophysiology of TBI, the focus is on the use of microRNAs as biomarkers and the diagnostic criteria for determining the severity of injury and/or the presence of injury in general.

One undoubtable advantage of this approach is that bodily fluids are more readily available and less invasive than biopsies. Although miR-20a is not a univocally nervous tissue specific miR, it can be used as a differentiating marker for the estimation of TBI severity. Bohemia and colleagues explored the diagnostic power of 10 miRNAs (miR-151-5p, miR-195, miR-20a, miR-328, miR-362-3p, miR-30d, miR-451, miR-486, miR-505, and miR-92a) in patients experiencing different levels of TBI [125]. It was demonstrated that miR-20a expression was upregulated in the serum and CSF of mild to moderate and severe TBI patients 2 days after injury. This miRNA can also help distinguish mild TBI cases from two non-TBI groups (healthy and orthopedic injury controls), which is very important for the diagnosis of asymptotic injury [126]. Moreover, Di Pietro et al. [127] showed an even less invasive method of diagnosis. Researchers collected the saliva samples from a well-characterized cohort of contact sport-professional and semiprofessional athletes, and demonstrated that several miRNAs, including hsa-miR-20a-5p and hsa-miR-20b-5p, can help to distinguish concussed athletes from non-concussed athletes after 48–72 h following injury [127].

## 6. Neurodegenerative Diseases

### 6.1. Alzheimer’s Disease

Alzheimer’s disease (AD) is the most widespread age-related dementia. It can overlap with other dementias, including vascular, Lewy body, and frontotemporal dementia. These all have no cure, no effective treatment, and only minor palliative care. In 2010, it was estimated that there were 35.6 million people living with dementia across the globe; these numbers are expected to double every 20 years until 2050 [2].

Pathologically, AD is characterized by the deposition of amyloid plaques [128] and tau tangles [129]. Plaque deposition is a consequence of the generation and aggregation of soluble amyloid β (Aβ) peptides. Once the Aβ peptide is generated, it can lead to neuronal apoptosis through the aberrant activation of plasma membrane expressed receptors, p75 neurotophin receptor [130,131] and N-methyl-D-aspartate receptor (NMDAR) [132]. An increase in deposition and a reduction in clearance of this peptide may play a key role in the disorder [133]. Aβ peptide is cleaved out of a large transmembrane amyloid precursor protein (APP) by two enzymes sequentially, β-site APP-cleaving enzyme-1 (BACE1) and the γ-secretase complex [134,135]. According to the amyloid cascade hypothesis [136], the elimination of amyloid plaque is expected to prevent or arrest AD progression, and, therefore, APP, BACE1, and γ-secretase have been selected as drug targets in the treatment of AD.

A novel study demonstrates that as few as 40 miRNAs are involved in the development of AD. One of the most thoroughly studied is miR-29a, which belongs to a miR family of the same name. It was proven that decreased levels of miR-29a/b-1 can promote Aβ production and can contribute to the pathogenesis of AD [76]. Subsequently, miR-29c, another miR-29 family member, was also found to be downregulated, with abnormally high levels of BACE1 in sporadic AD brains. Another well studied miRNA is miR-107. The expression of miR-107 decreased significantly, with BACE1 increased in AD patients. Nelson and Wang also demonstrated that miR-107 levels negatively correlated with BACE1 mRNA levels, leading to Aβ accumulation [77]. It has been reported by many groups that miR-132 is decreased significantly in AD brains. Zhao et al. [78] recently discovered that the overexpression of miR-132 in cultured cortical neurons could inhibit the neurotoxicity induced by Aβ via the miR-132/PTEN/AKT/FOXO3a pathway. A new pathway, miR-124/ protein-tyrosine phosphatase 1 (PTPN1), has recently been reported to be involved in synaptic transmission deficits. The level of miR-124 increased significantly with the decrease in expression of its target, PTEN1, in AD brains. Moreover, the inhibition of miR-124 expression or the over-expression of PTPN1 could alleviate the synaptic deficits in AD model mice [78].

The first evidence that APP expression is regulated and can be manipulated by miRNAs was provided by Hebert et al. [76]. They demonstrated that the overexpression of miR-20a can regulate the expression of endogenous APP protein in cell lines in vitro. APP expression regulation by miRNA was observed in different cell types, including mouse and human neuroblastoma cell lines. The remarkable correlation in miR-20a expression (together with miR-17-5p/106b) with APP levels was observed during brain development, and in differentiating neurons in vitro. More than ten years later, two different groups independently showed that miR-20b-5p plays an important role in the development of AD [76].

**Figure 5:**
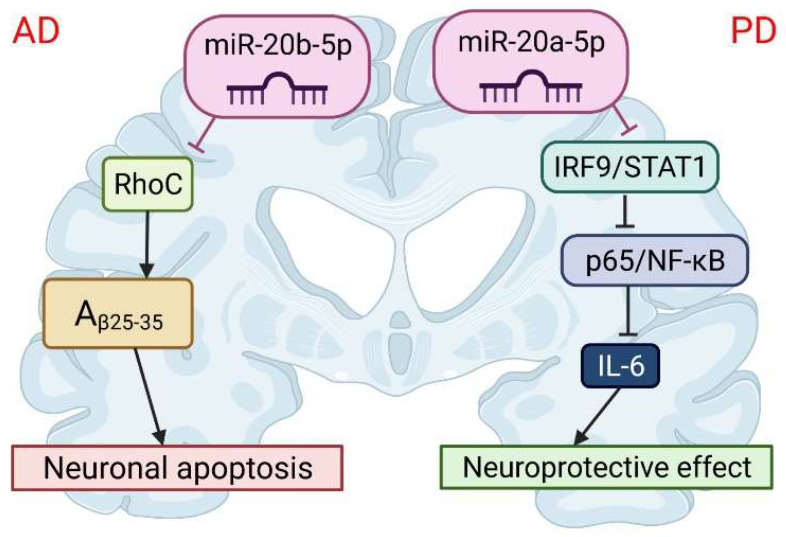
The role of miR-20 in the pathophysiology of neurodegenerative diseases. miR-20b-5p could disrupt Alzheimer’s disease progression by regulating neuronal apoptosis and cell viability by targeting the RhoC gene. miR-20a-5p has a protective effect on Parkinson’s disease-related neuronal death, which can be achieved by targeting the IRF9/NF-κB axis. This image was created using BioRender (accessed on 30 March 2022).

Tian et al. [79] investigated the expression of miR-20b-5p and target RhoC in the brains of APPswe/PSΔE9 mice (Figure 5). The initial characterization of this mouse line indicated a progressive increase in amyloid beta peptide deposition. RhoC, also known as Rho-Related GTP-binding Protein RhoC, belongs to the Rho family [137], and is implicated in NSC’s migration [138], cell morphology and function, axon regeneration, and immunity through many signaling pathways [139]. The expression of miR-20b-5p was increased, and the expression of RhoC was decreased in the hippocampus of APPswe/PS△E9 mice. They also demonstrated, on PC12 cells, that the knockdown of RhoC aggravated the inhibition effect on cell viability induced by Aβ25−35, while the miR-20b-5p inhibitor diminished these effects. In conclusion, the inhibition of miR-20b-5p attenuates apoptosis induced by Aβ25−35 in PC12 cells through the targeting of RhoC.

A similar conclusion was made by Wang et al. [140], who identified miR-20b as a negative regulator of APP in human cell lines and in primary human brain cell culture. They further showed that elevated miR-20b is associated with a greater risk of AD in post-mortem brains, and also that miR-20b’s reduction of APP expression was reversed by the addition of an antagonistic miR to miR-20b. Furthermore, the Wang group reported that miR-20b can disrupt calcium homeostasis, neurite outgrowth, and neuronal branchpoints in a primary human cell culture model. Therefore, miR-20b-5p may be a perspective curative target for AD [140].

### 6.2. Parkinson’s Disease

Parkinson’s disease (PD) is the second most common neurodegenerative disease after Alzheimer’s disease, affecting approximately 6 million people worldwide [141]. PD involves progressive and irreversible loss of dopaminergic neurons in the brain, especially in the substantia nigra (SN). When more than 50–70% of dopamine neurons are lost, it also leads to a reduction in the production and signaling of the dopamine neurotransmitters [142]. The loss of dopaminergic neurons leads to various motor dysfunctions, such as tremor bradykinesia rigidity and postural instability [143]. Numerous mechanisms are associated with the pathophysiology of PD, such as α-synuclein accumulation, mitochondrial dysfunction, oxidative stress, calcium homeostasis, and neuroinflammation [144]. Intracellular inclusions known as Lewy bodies, which are enriched in the aggregated protein α-synuclein, are also frequently found in the neurons of PD patients, and are thought to interfere with pathways such as vesicle transport or neuroinflammation activation [145]. MicroRNA analysis in PD has identified several miRNAs that are consistently expressed differently in the blood and brain of patients with PD [146]. One of the most brain-specific miRNAs, miR-124, has been found to be downregulated in a mouse SN (MPTP-induced model of PD) along with increased levels of calpain/ cyclin dependent kinase 5 proteins [80], thus targeting the neuroinflammation processes related to PD. The members of the miR-34 family along with miR-126, affect the survival of dopaminergic neurons. A functional role for increasing miR-126 levels in SN dopaminergic neurons has been shown in PD patients through the inhibition of the IGF-1/PI3K signaling pathway, which contributes to neurotoxicity [81]. miR-34b and c levels were found to be significantly downregulated in the amygdala, frontal cortex, cerebellum, and spinal cord of PD patients, accompanied by a decrease in PARK2 and PARK7 expression. In addition, they were involved in changes in mitochondrial function and oxidative stress [82] and, together with miR-95, contributed to the decrease in the expression of α -synuclein DJ-1 [147] and Parkin [83], while miR-95 regulated the lysosomal function through the sulfatase-modifying factor 1 enzyme [84]. In post-mortem human brain studies, let-7 family members were found to be upregulated in PD patients when compared to healthy controls. They also promote polarization of macrophages from the M1 phenotype to the M2 phenotype and can act as endogenous damage-associated molecular patterns and are recognized by toll-like receptor 7 (TLR7), promoting inflammation and neuronal death [148].

Recent studies have demonstrated that miR-20a-5p also contributes to neuroinflammation and oxidative stress and can serve as a novel therapeutic target for PD [149]. Wang et al. [149] has shown that miR-20a-5p alleviated mitochondrial dysfunction, inflammation, and cell apoptosis, induced by 1-methyl-4-phenyl pyridine ion- (MPP+)—an in vitro model of PD (Figure 5). This was realized by targeting interferon regulatory factor 9 (IRF9). IRF9 is a member of a family of interferon regulatory factors and plays an important role in antivirus, immune response, cell growth regulation, and apoptosis. Moreover, STAT1/IRF9 complex causes a pro-inflammatory effect by regulating the transcription of the CXCL10 gene [85]. Simultaneously, the STAT1/IRF9 complex could bind to the p65 subunit of NF-κB and result in increased synthesis of IL-6 [150]. In this study, IRF9 hindered the improvement of miR-20a-5p overexpression on MPP+-induced neurotoxicity. Furthermore, the decrease of p-P65 level induced by miR-20a-5p mimic was significantly reversed by IRF9 overexpression. Thus, miR-20a-5p has a protective effect on neuronal death related to PD. The neuroprotective effect of miR-20a-5p was achieved in part by targeting the IRF9/NF-κB axis.

## 7. Conclusions

There is compelling evidence that clearly demonstrates an association between the dysregulation of miRNAs, miRNA-20 in particular, and neurological disorders. While some pathological conditions, such as SCI or AD, are accompanied by an increase in miR-20 expression and, accordingly, their correction involves the inhibition of miR-20 activity, others, stroke and PD, lead to more complex or opposite changes in miR-20 expression. These contradictions can be explained by such aspects of microRNA biology as a multi-target effect, the ability to act in a coordinated manner or the cellular context of its expression. Further research is needed to elucidate its mechanism of action and determine the most appropriate therapeutic window for treating specific diseases. miRNA-based therapies have become one of the most promising strategies for the treatment of incurable neurological disorders. However, to increase their effectivity, it is necessary to develop pharmacological formulations and delivery methods that can cross the BBB into brain tissues, as well as develop methods to reduce off-target effects. Despite numerous attempts to develop miRNA-based therapeutics, none of them have been approved, for example, by the FDA. This requires a good knowledge of miRNA target genes and a network that can help develop alternative therapeutic plans.

## Figures and Tables

**Table 1:** The most canonical microRNAs and their validated targets, which are dysregulated due to neurological disorders (ND). SCI—spinal cord injury, SDLC—spinal dorsal column lesion, TBI—traumatic brain injury, AD—Alzheimer’s disease, TPM1—tropomyosin alpha-1 chain, PTEN—phosphatase and tensin homolog, PDCD4—programmed cell death protein 4, TNF-α—tumor necrosis factor alfa, IL-6—interleukin-6, IL-1β—interleukin-1 β, COX2—cyclooxygenase-2, STAT3—signal transducer and activator of transcription 3, NRF2—nuclear factor erythroid 2-related factor 2, Aβ—amyloid beta, BACE1—beta-secretase 1, protein-tyrosine phosphatase 1 – PTPN1, IGF-1/PI3K—insulin growth factor/phosphoinositide 3-kinases, PARK—parkin, SUMF1—sulfatase-modifying factor 1, IRF9—interferon regulatory factor 9.

ND	Dysregulated microRNAs	Validated Targets	References
SCI	miR-21	Fas-ligand, TPM1, PTEN, PDCD4	[54,55,56]
	miR-181, miR-125b	TNF-α	[58,59]
	let-7a, miR-181a, miR-30b-5p, miR-30c	IL-6 and IL-1β	[50,52,59,60]
	miR-133b	RhoA	[61]
	miR-124, miR-34a, miR-219	Syntaxin-1A, synaptotagmin-1, p53	[62]
	miR-20a	Neurogenin1, IL-6, IL-1β, TNF-α, COX2, caspase-3, STAT3, Mcl-1	[51,63]
SDLC	miR-20a	GTP-RhoA, Nr4a3	[64,65]
Stroke	miR-124	JAG-Notch signaling	[66,67]
	miR-145	Superoxide dismutase-2	[4]
	miR-497	Bcl-2, Bcl-w	[68]
	miR-15a	Bcl-2	[69]
	miR-320a	Aquaporins	[70]
	miR-21	Fas-ligand	[54]
	miR-20a	NeuroD1	[71]
TBI	miR-107	Granulin	[72]
	miR-34a	Notch1	[73]
	miR-144	Cask, NRF2	[74]
	miR-23a and miR-27a	Bcl-2	[75]
AD	miR-29a/b-1	Aβ	[76]
	miR-29c, miR-107	BACE1	[76,77]
	miR-132	PTEN	[77]
	miR-124	PTPN1	[78]
	miR-20a-5p	RhoC	[79]
PD	miR-124	Calpain/CDK5	[80]
	miR-34, miR-126	IGF-1/PI3K	[81]
	miR-34b	PARK2, PARK7	[82]
	miR-95	α-synuclein, Parkin, SUMF1	[83,84]
	miR-20a-5p	STAT1/IRF9	[85]
